# Perception of personalized medicine, pharmacogenomics, and genetic testing among undergraduates in Hong Kong

**DOI:** 10.1186/s40246-021-00353-0

**Published:** 2021-08-18

**Authors:** Nicholas Yan Chai Cheung, Jasmine Lee Fong Fung, Yvette Nga Chung Ng, Wilfred Hing Sang Wong, Claudia Ching Yan Chung, Christopher Chun Yu Mak, Brian Hon Yin Chung

**Affiliations:** 1 Bachelor of Medicine and Bachelor of Surgery Program, Li Ka Shing Faculty of Medicine, The University of Hong Kong, Hong Kong, SAR China; 2 Department of Paediatrics and Adolescent Medicine, Li Ka Shing Faculty of Medicine, The University of Hong Kong, Hong Kong, SAR China; 3 Department of Paediatrics and Adolescent Medicine, Queen Mary Hospital, Hong Kong, SAR China; 4 Department of Paediatrics and Adolescent Medicine, Hong Kong Children’s Hospital, Hong Kong, SAR China

**Keywords:** Education, Ethical, Legal and Social Implications, Genetic testing, Personalized medicine, Pharmacogenomics

## Abstract

**Background:**

The global development and advancement of genomic medicine in the recent decade has accelerated the implementation of personalized medicine (PM) and pharmacogenomics (PGx) into clinical practice, while catalyzing the emergence of genetic testing (GT) with relevant ethical, legal, and social implications (ELSI).

**Results:**

The perception of university undergraduates with regards to PM and PGx was investigated, and 80% of undergraduates valued PM as a promising healthcare model with 66% indicating awareness of personal genome testing companies. When asked about the curriculum design towards PM and PGx, compared to undergraduates in non-medically related curriculum, those studying in medically related curriculum had an adjusted 7.2 odds of perceiving that their curriculum was well-designed for learning PGx (95% CI 3.6–14.6) and a 3.7 odds of perceiving that PGx was important in their study (95% CI 2.0–6.8). Despite this, only 16% of medically related curriculum undergraduates would consider embarking on future education on PM.

When asked about their perceptions on GT, 60% rated their genetic knowledge as “School Biology” level or below while 76% would consider undergoing a genetic test. As for ELSI, 75% of undergraduates perceived that they were aware of ethical issues of GT in general, particularly on “Patient Privacy” (80%) and “Data Confidentiality” (68%).

Undergraduates were also asked about their perceived reaction upon receiving an unfavorable result from GT, and over half of the participants perceived that they would feel “helpless or pessimistic” (56%), “inadequate or different” (59%), and “disadvantaged at job seeking” (59%), while older undergraduates had an adjusted 2.0 odds of holding the latter opinion (95% CI 1.1–3.5), compared to younger undergraduates.

**Conclusion:**

Hong Kong undergraduates showed a high awareness of PM but insufficient genetic knowledge and low interest in pursuing a career towards PM. They were generally aware of ethical issues of GT and especially concerned about patient privacy and data confidentiality. There was a predominance of pessimistic views towards unfavorable testing results. This study calls for the attention to evaluate education and talent development on genomics, and update existing legal frameworks on genetic testing in Hong Kong.

**Supplementary Information:**

The online version contains supplementary material available at 10.1186/s40246-021-00353-0.

## Background

The advancement of genomic medicine in recent decades has brought major breakthroughs in the healthcare system [1–3]. Initial efforts like the completion of the Human Genome Project to subsequent projects like the Exome Aggregation Consortium (ExAC) and the Genome Aggregation Database (gnomAD) have elucidated genetic variations between individuals via databases of ancestry-based genetic variants [4–9]. In parallel, the maturation and adoption of high-throughput genomic technologies such as the next-generation sequencing (NGS) technique for whole genome sequencing and whole exome sequencing allowed more rapid diagnosis of genetic conditions and discovery of novel genes for polygenic diseases [10–15].

As these state-of-the-art genetic and genomic technologies are integrating to our healthcare system in the form of genetic testing (GT), their applications are becoming more accessible, extending to direct-to-consumer genetic testing (DTCGT) in recent years [16, 17]. DTCGT are more consumer-oriented and readily accessible, where consumers enjoy high autonomy from test initiation to genetic information management; in contrast to traditional clinical genetic and genomic testing [18]. With the provision of a spectrum of GT including pharmacogenomic tests, individualized yet enormous volume of medical information is generated. In parallel, an array of relevant ethical, legal, and social implications (ELSI) have surfaced, especially on DTCGT, ranging from data protection to clinical utility [19, 20]. All of these issues have prompted controversies and discussions among the public amid the rapid and widespread provision of DTCGT [16, 21, 22].

Personalized medicine (PM) refers to the process of tailoring medical services, including prevention and treatment, to individuals based on their biological features such as susceptibility to diseases and responses to drugs [23, 24]. In the era of PM, the incorporation of genomic information to clinical data has improved, optimizing medical care provided to each individual [25]. The uniqueness of each human genome has enabled the application of genomic data to individualize services and improve outcomes, and in the research field, to discover genes for rare diseases or even gene therapy for previously incurable diseases [26–29]. Both clinical and research outcomes possess the potential to feedback with each other, creating a virtuous cycle between “bedside” and “bench,” thereby promoting the implementation of PM [30–32].

Pharmacogenomics (PGx), one of the clinical applications of PM, studies the optimization of drug efficacy and dosage, and the minimization of adverse drug reactions (ADR) based on variations and alterations in the genome of each individual [33–36]. The emergence of NGS has facilitated the development and enrichment of databases such as The Pharmacogenomics Knowledge Base (PharmGKB) and The Clinical Pharmacogenetics Implementation Consortium (CPIC) [37]. These databases have not only organized pharmacogenomic information in a more systematic, standardized, and evidence-based manner, they have also provided clinical recommendations including gene-drug-disease relationships; all of which have fostered the transfer of pure pharmacogenomic knowledge into daily practices and contributed to the development of personalized medicine [38, 39].

The visions of future healthcare are the maturation of PGx and PM in the healthcare system and the widespread application of GT in our society [40, 41]. However, numerous challenges lie ahead, notably education of the next-generation “service providers” with expertise in PGx and PM as well as the public on the essentials of genetics and genomics [42–44]. Education of both stakeholders play an indispensable role in the application of genomic medicine in the society [45–47]. In response to the challenge, various transnational organizations have been promoting the incorporation of PGx and PM in medical education; in addition to national public education frameworks [48–50]. The evaluation of PGx and PM education has shown that most medical and pharmacy schools have incorporated them into their courses [51, 52]. In particular, a global study demonstrated that 87% of the responded medical and pharmacy schools have PGx and PM education [53]. On the contrary, investigations on public understanding of genetics and genomics have revealed a worrying situation. The general public, without any medical background, demonstrated insufficient knowledge on genetic and genomics in several studies. The pattern was similar even in undergraduates studying in non-science majors, whom are regarded as a group of individuals having relatively higher educational level among the general population [54, 55].

In addition to the assessment of PGx and PM coverage in undergraduate education, recent studies also evaluated the perception and awareness of undergraduates on related concepts, with most undergraduates having a positive attitude towards PGx and PM [56–59]. These studies served as the groundwork for educational bodies on revising curriculum maps; and for the national and international community to determine the future direction of implementing PGx and PM into public education and clinical practice.

In Hong Kong, the clinical applications of PGx and PM have been brought under public attention with the establishment of the Hong Kong Genome Project (HKGP) [60]. However, there are currently very few local studies on public awareness and perception on PM, PGx, and GT, or the coverage of PM and PGx education at the undergraduate level. These are important as they could serve as a reference for policymaking and curriculum design. Therefore, this study aims to investigate (i) perception and education on PM and PGx; and (ii) perception on GT and relevant ELSI among undergraduates in Hong Kong.

## Methodology

This cross-sectional study was conducted using online questionnaires between February and April in the academic year 2018 to 2019 and 2019 to 2020 respectively in the University of Hong Kong (HKU). The questionnaire was accessed online at the commencement of the University’s Common Core Course “The World Changed by DNA” by HKU undergraduates by convenience sampling, of which students from any study curriculum in HKU could register for this course. Undergraduates enrolled in this course were either from medically related curriculum (MRC) (Biomedical Sciences, Chinese Medicine, Dentistry, Medicine, Nursing, Pharmacy) or non-medically related curriculum (NMRC) (Arts, Business and Economics, Education, Engineering, Law, Social Science, and Others). Participants were required to complete an online questionnaire, which was designed and modified based on a questionnaire from a study published by Mahmutovic et al., to investigate the perception of undergraduates in health and molecular life sciences on PGx and PM [61]. G*power version 3.1.9.7 software was used to estimate sample size. Based on the data of Mahmutovic et al., we estimated that the minimum percentage difference in genetic testing attitudes between two groups was around 28% [61]. For 80% power at 5% level of significance with two tails, a minimum of 52 subjects in each curriculum group (*n* = 104) is required to reach a significant result. Participants were assured that their personal identifiers remain confidential. The participation in this study was voluntary and informed consent was obtained. Ethics approval was granted by the Institutional Review Board, the University of Hong Kong/Hospital Authority Hong Kong West Cluster (UW 19-609).

The questionnaire consisted of 32 questions, which were divided into four sections: (i) demographics (age, gender, and field of study), (ii) perception of PM and PGx, (iii) education on PM and PGx, and (iv) awareness on GT and relevant ELSI. All categorical responses were expressed as frequencies and percentages. Data from our study was stratified into two groups according to students’ curriculum—MRC and NMRC. Responses other than “Yes” or “Agree” were grouped together as “Negative” for statistical analysis.

Fisher’s exact test and Chi-Square test were performed on categorical variables for descriptive analysis. Binary logistic regression was performed to investigate the association of the questions of interest and covariates including curriculum, age and gender, where age was grouped into “< 19 years old” and “≥ 19 years old”. Odds ratio (OR) and corresponding 95% confidence intervals (CI) were computed with the level of significance set at *p* < 0.05. Statistical analysis was performed by IBM SPSS Version 26.

## Results

### Participants’ demographics

Between February and April in 2018 and 2020, 231 undergraduates were recruited from the University’s Common Core Course “The World Changed by DNA”; 202 completed the survey and provided informed consent for data collection for research purpose. The characteristics of the participants are summarized in Table 1. Demographic characteristics were reasonably well balanced with no statistical difference between students in the MRC and NMRC. Among the 202 participants, 81 were undergraduates studying in MRC and 121 were undergraduates studying in NMRC. Majority of undergraduates were female (66%), studying in year 1 (61%) and below 21 years old (96%).

**Table 1:** Undergraduates’ demographics and curriculum

	Overall (*n* = 202)	Medically related curriculum (*n* = 81)	Non-medically related curriculum (*n* = 121)
**Gender**			
Male	68 (34%)	26 (32%)	42 (35%)
Female	134 (66%)	55 (68%)	79 (65%)
**Year of Study**			
Year 1	123 (61%)	56 (69%)	67 (55%)
Year 2	66 (33%)	22 (27%)	44 (36%)
Year 3	12 (6%)	3 (4%)	9 (7%)
Year 4	1 (0%)	0 (0%)	1 (0%)
**Age**			
< 19	89 (44%)	40 (49%)	49 (40%)
19– 21	105 (52%)	37 (46%)	68 (56%)
21– 23	6 (3%)	2 (2%)	4 (3%)
> 23	2 (1%)	2 (2%)	0 (0%)

### Perception and education on personalized medicine and pharmacogenomics

**Fig. 1:**
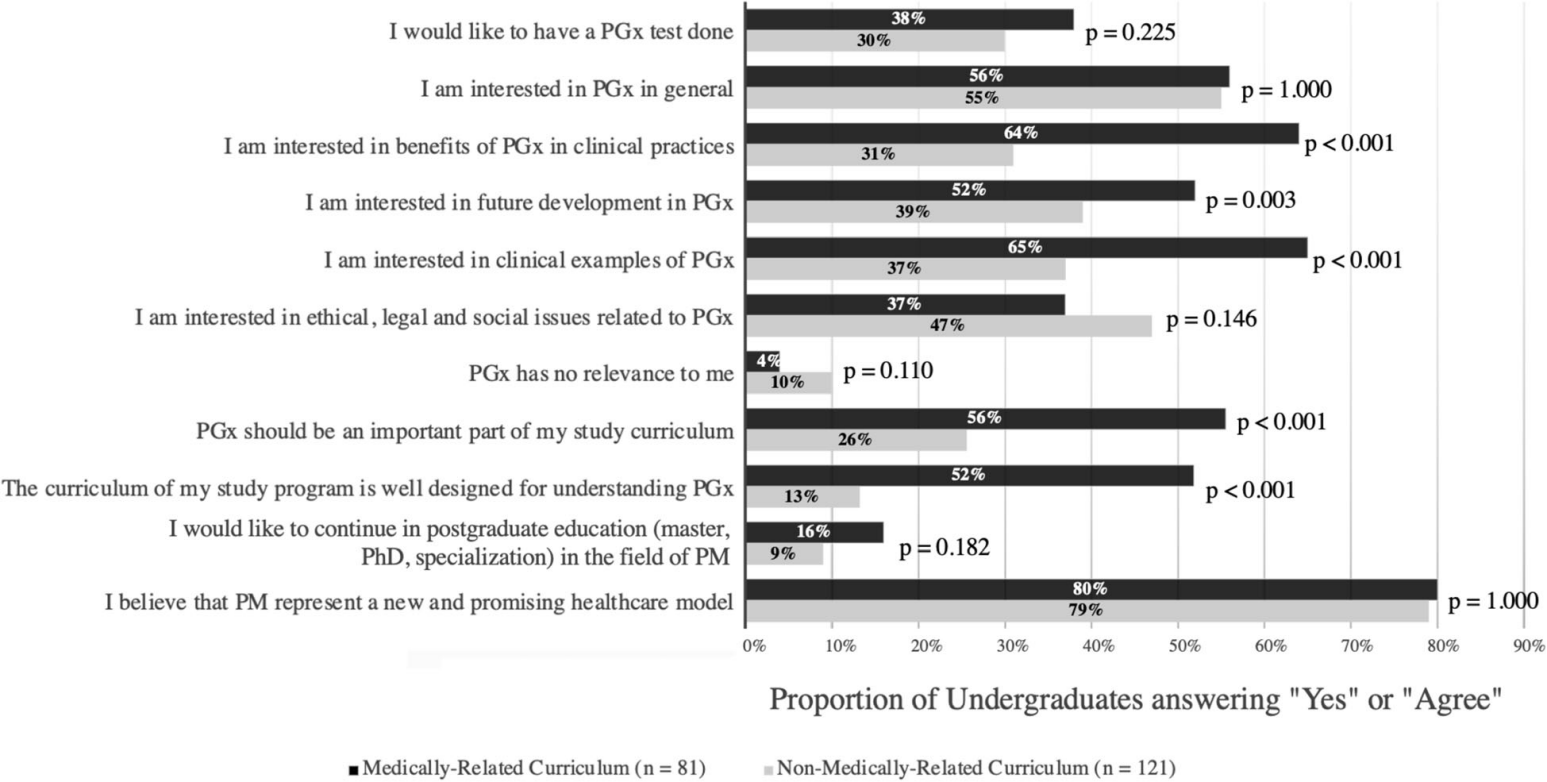
Undergraduates’ perception and education on personalized medicine and pharmacogenomics Proportion of MRC and NMRC undergraduates answering “Yes” or “Agree” in each statement, with the level of significance between two groups presented as *p* values for each question. *PGx* pharmacogenomics, *PM* personalized medicine

In total, 80% of undergraduates (161/202) agreed that PM is a promising new healthcare model, regardless of curriculum (*p* = 1.000) (Fig. 1). Regarding PGx testing, 47% of undergraduates (95/202) were uncertain whether they would be interested in doing a PGx test or not, while only 33% of undergraduates (67/202) would like to have it done. The pattern was similar among MRC and NMRC undergraduates (*p* = 0.225).

In general, 56% (45/81) of MRC undergraduates perceived that PGx should be important to their curriculum and 52% (42/81) agreed that their curriculum was well-designed for PGx, compared to 26% (31/121) and 13% (16/121) of NMRC undergraduates respectively (Fig. 1). The differences observed between MRC and NMRC undergraduates were both significant at *p* < 0.001. As shown in Table 2, undergraduates studying in MRC had an adjusted 3.7 odds (95% CI 2.0–6.8, *p* < 0.001) of perceiving that PGx was important and 7.2 odds (95% CI 3.6–14.6, *p* < 0.001) of their curriculum was well designed for PGx, comparing to NMRC undergraduates.

**Table 2:** Association between perception and education on personalized medicine and pharmacogenomics and demographics

	Adjusted OR	95% CI	*P* value
Pharmacogenomics (the interaction between drugs and genetics) should be an important part of my study curriculum			
Medically related curriculum	3.721	2.027–6.829	< 0.001
≥ 19 years old	0.936	0.509–1.720	0.831
Male	1.653	0.878–3.115	0.120
Do you think that curriculum of your study program is well designed for understanding pharmacogenomics?			
Medically related curriculum	7.246	3.608–14.550	< 0.001
≥ 19 years old	0.594	0.299–1.181	0.137
Male	1.801	0.878–3.693	0.108
**Would you like to continue your postgraduate education (master, PhD, specialization) in the field of personalized medicine?**			
Medically related curriculum	1.878	0.793–4.445	0.152
≥ 19 years old	0.802	0.338–1.906	0.618
Male	1.027	0.411–2.564	0.955

*CI* confidence interval, *OR* odds ratio

Among different PGx topics, undergraduates were most interested in “Pharmacogenomics in general” (55%), followed by “Clinical examples of pharmacogenomics” (49%). Only 7% of undergraduates regarded PGx irrelevant to them. However, merely 12% (24/202) of undergraduates would consider postgraduate education in PM with the pattern similar in MRC and NMRC undergraduates (*p* = 0.182). The perception was independent of curriculum, age, and gender.

### Perception on direct-to-consumer genetic testing and relevant ethical, legal and social implications

**Fig. 2:**
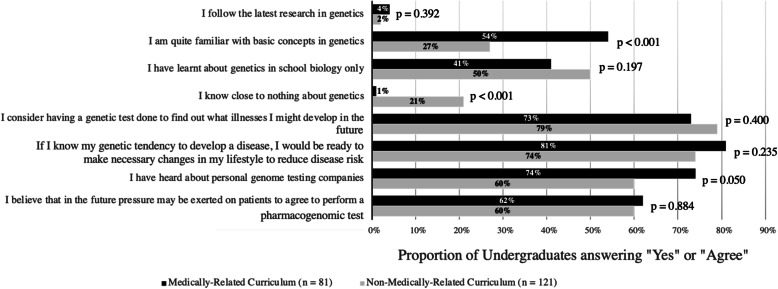
Undergraduates’ perception on direct-to-consumer genetic testing Proportion of MRC and NMRC undergraduates answering “Yes” or “Agree” in each statement, with the level of significance between two groups presented as p-values for each question

Among the 202 undergraduates, 60% (120/202) regarded their genetic literacy as school biology level or below, while only 2% (5/202) would follow the latest research in genetics (Fig. 2). Overall, 76% (154/202) of undergraduates would like to have a GT done to discover possible illnesses they might develop in the future, regardless of curriculum (*p* = 0.400). Additionally, if a genetic tendency to develop a disease is revealed, 77% (155/202) of undergraduates were ready to make necessary changes to their lifestyle to reduce the risk, irrespective of curriculum (*p* = 0.235). Moreover, 66% (133/202) of undergraduates had heard of personal genome testing companies, indifferent between MRC and NMRC undergraduates (*p* = 0.050). Lastly, 61% of undergraduates (123/202) held the perception that future pressure would be exerted on patients to perform a PGx test, indifferent between MRC and NMRC undergraduates (*p* = 0.884).

**Fig. 3:**
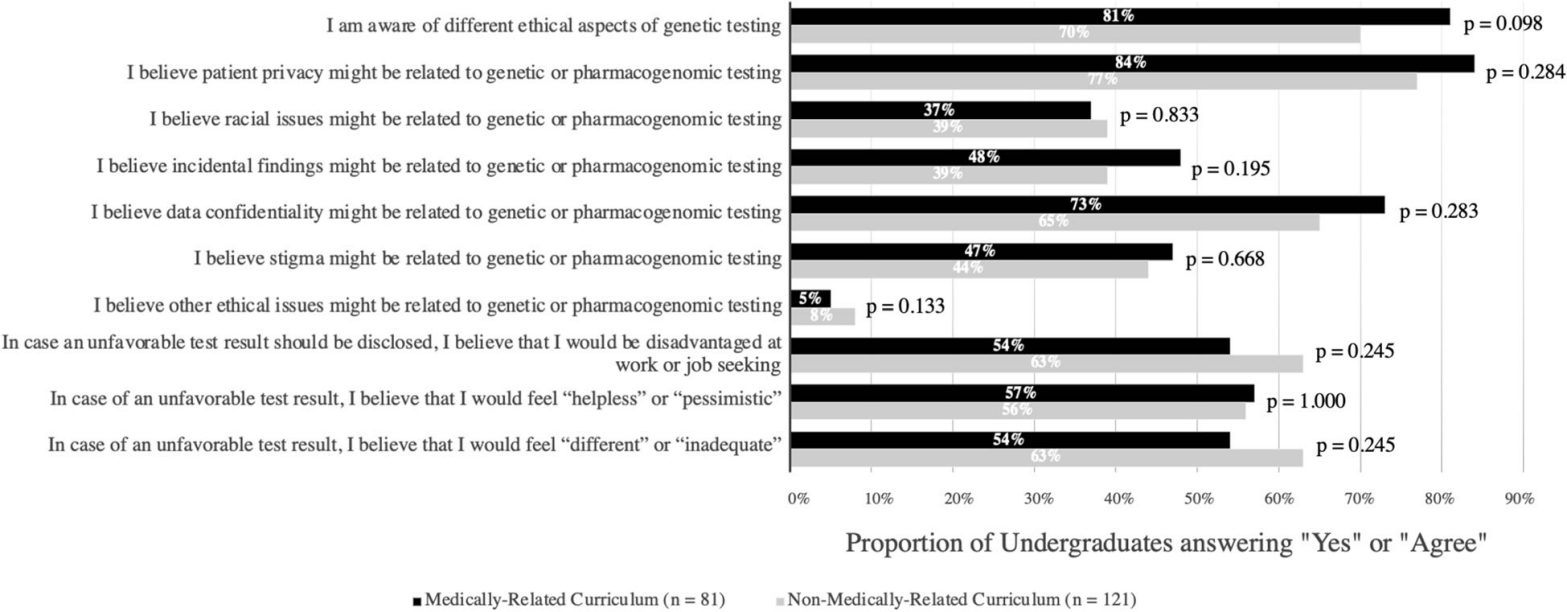
Undergraduates’ perception on ethical, legal and social implications related to direct-to-consumer genetic testing Proportion of MRC and NMRC undergraduates answering “Yes” or “Agree” in each statement, with the level of significance between two groups presented as p-values for each question

Among the 202 undergraduates, 75% (151/202) perceived that they were aware of the ethical aspects  of GT, and the patterns were similar across MRC and NMRC undergraduates (*p* = 0.098) (Fig. 3). Out of the five suggested ethical issues, the median number of issues perceived as related to genetic or PGx testing by both MRC and NMRC undergraduates was three. Majority of undergraduates agreed that “Patient Privacy” (80%) and “Data Confidentiality” (68%) were ethical issues related to genetic or PGx testing, while only less than half of the undergraduates regarded “Stigma” (45%), “Incidental Findings” (43%), “Racial Issues” (38%), and “Others” (7%) as ethical issues related to genetic or PGx testing.

In relation to the perception of an unfavorable genetic test result, majority of undergraduates would feel disadvantaged at job seeking (59%), helpless or pessimistic (56%), and inadequate or different (59%). No differences were observed between MRC and NMRC undergraduates (*p* = 0.245, 1.000, 0.245). While the perception of pessimism and inadequacy upon unfavorable results were independent of curriculum, age, and gender, older undergraduates (≥ 19 years old) had an adjusted 2.0 odds of holding the perception of feeling disadvantaged at job seeking than younger undergraduates (< 19 years old) (95% CI 1.1–3.5, *p* = 0.023) (Table 3).

**Table 3:** Association between perception and education on personalized medicine and pharmacogenomics and demographics

	Adjusted OR	95% CI	*P* value
In case an unfavorable test result should be disclosed, do you believe that you would be disadvantaged at work or job-seeking?			
Medically-related curriculum	0.731	0.408–1.309	0.292
≥ 19 years old	1.957	1.097–3.490	0.023
Male	0.676	0.369–1.238	0.205
In case of an unfavorable test result, do you believe that you would feel “helpless” or “pessimistic”?			
Medically related curriculum	1.026	0.579–1.819	0.931
≥ 19 years old	1.146	0.650–2.019	0.638
Male	0.667	0.370–1.204	0.179
In case of an unfavorable test result, do you believe that you would feel “different” or “inadequate”?			
Medically related curriculum	0.681	0.381–1.215	0.194
≥ 19 years old	0.840	0.472–1.497	0.555
Male	0.611	0.337–1.110	0.106

*CI* confidence interval, *OR* odds ratio

## Discussion

### Hong Kong undergraduates’ learning on pharmacogenomics and career development of personalized medicine

Our results demonstrated 80% of undergraduates regarded PM as a promising healthcare model and 76% would consider having a genetic test done. Nevertheless, in terms of undergraduates’ own studies and future planning on PM and PGx, less than 60% of undergraduates, even in the MRC, believed that PGx is important and that their curriculum was well-designed for understanding PGx. Undergraduates with academic background of MRC had higher odds of holding the two opinions, adjusted for age and gender, which aligns with the pattern that PM and PGx are usually included in MRC than NMRC education due to higher relevance to their learning and future practice as healthcare professionals [62, 63].

Furthermore, only 16% of MRC undergraduates would consider continuing with postgraduate education on PM, suggesting a shortfall of PGx education and talent development in PM in Hong Kong. Local education and nurturing of experts in PM and PGx have been known to be inadequate in Hong Kong, as reflected by the delivery of genomic education at a later learning stage compared to the USA [60, 64]. Local education on genetics and genomics starts at secondary school level, while in other Western countries such as the USA, education on related concepts is initiated at kindergarten to primary school level [48, 65]. In the USA, basic concepts of heredity are introduced to kindergarteners with the use of common examples, such as cats delivering kittens with different markings, thus illustrating the variation of traits [66]. Hence, the education curriculum in the USA demonstrated the possibility of implementing genomic education and establishing a robust foundation on genomics at an early learning stage.

The general situation of insufficient education and talent development on PM and PGx might be exaggerated by the delayed local development of PM and PGx until recent years. Compared to other Western countries, Hong Kong’s practice of PGx is still in its infancy. In 2019, the provision of PGx testing in Hong Kong public hospitals was only limited to three drugs; in contrast, current clinical application of PGx in other countries is much more extensive with wider gene-drug coverage [67]. For instance, in the Netherlands, up to July 2020, more than 100 gene-drug pairs were examined by the Dutch Pharmacogenetics Working Group (DPWG) including 60 actionable gene-drug pairs [68]. In Austria, the clinical application of the Medial Safety Code, which is a QR code encompassing personal PGx information on 54 drugs, has been explored since 2013 [69]. With Hong Kong’s development of PM and PGx lagging behind the global trend, it is important to enhance public engagement and nurture talents in genomic medicine, as highlighted as two of the eight recommendations in the plan of strategic development of genomic medicine in Hong Kong [60].

In recent years, the emergence of DTCGT companies have provided substantial information on PM and GT and emphasized the striking merit of genomic medicine to individual healthcare management [70]. However, undergraduates’ awareness of GT, PM, and PGx might be based mostly on the commercial advertisements and promotions on DTCGT which encompass misinformation and overemphasis, rather than stemming from more accurate knowledge from their undergraduate curriculum [71]. As a result, without an in-depth understanding of PM, PGx, and GT, it is challenging for undergraduates to develop a strong interest in further exploration of such topics, as reflected by the low proportion of undergraduates planning to continue postgraduate education in PM.

Therefore, the devotion of more attention and resources toward university education and career planning on PM and PGx possesses great potential to promote genomic medicine through a robust foundation of expertise and public support.

### Negative attitude to unfavorable results of direct-to-consumer genetic testing related to ethical, legal, and social implications

In our cohort, more than 50% of Hong Kong undergraduates displayed a negative attitude in case of an unfavorable result from GT, such as feeling “helpless or pessimistic,” “different or inadequate,” and “disadvantaged at job seeking” with undergraduates ≥ 19 years old had higher odds of holding the latter opinion. A similar association was also observed in a study where older adults were more worried on health compared to younger adults utilizing the Worry Scale for Older Adults [72]. The age-related perception that unfavorable GT results cast disadvantages in career pathway might be explained by the following two reasons. First, the fact that older undergraduates are closer to graduation may make them more anxious about any negative impacts on their future. Second, in specific to career planning, older undergraduates are closer to facing job applications; hence, they would be more aware of the issues potentially unfavorable to their competitiveness, such as that on insurance. A high level of concern over the above issues have been previously reported from a local study on Hong Kong adults on DTCGT regarding several areas, where they were more apprehensive on the lack of local regulation on DTCGT (78%), possible psychological harm (63%), and stigmatization (59%) [73]. Similar concerns have also been demonstrated in another local qualitative study [74]. They showed that Hong Kong undergraduates were concerned about genetic discrimination at workplace or even in educational institutions. In their thematic analysis, some undergraduates stated that employers tend to recruit excellent employees and upon discovering an unfavorable GT result, employers might provide fewer resources on training that employee, and are less willing to spend on resources to train up employees upon discovering unfavorable GT results.

In contrast, undergraduates in Bosnia and Herzegovina shared a more optimistic view on ELSI in case of unfavorable testing results. Using the same set of questions, only less than one-third of undergraduates in the study by Mahmutovic et al. displayed a negative attitude, in contrast to over 50% in Hong Kong. Similarly, a recent study on 346 undergraduates in Greece has demonstrated that students were very optimistic about the usefulness of GT, and professed positive anticipations on PGx for disease management [59]. The trend of pessimism among local undergraduates could be potentially explained by the phenomenon that Asians are more pessimistic in general as compared to other ethnic groups. A study published by Chang et al. demonstrated that Asian Americans were more pessimistic than Caucasian Americans in general [75]. Similarly, another study published by Lee et al. also illustrated that mainland Chinese students were more pessimistic than Chinese American students, and Chinese American students were more pessimistic than Caucasian American students [76]. While the trend of pessimism on unfavorable GT results is particularly prominent and general among Hong Kong undergraduates, the underlying reasons of pessimism as well as possible measures to reverse the trend should be pondered on and properly addressed.

The pattern that more Hong Kong undergraduates were worried about genetic discrimination at the workplace comparing to the study by Mahmutovic et al. might be brought about by the slow development of a legal doctrine on anti-genetic discrimination compared to the global trend [61]. One of the most well-known and early established legal frameworks in terms of safeguarding citizens’ rights and interests against ELSI in GT is the Genetic Information Non-discrimination Act (GINA) of 2008 in the USA. The European Union Charter of Fundamental Rights Article 21 and the Convention on Human Rights and Biomedicine also serve similar purposes in the prohibition of genetic discrimination [77]. However, in Hong Kong, the Disability Discrimination Ordinance and the Personal Data (Privacy) Ordinance were not regarded as comprehensive or specific as the aforementioned foreign legal frameworks to prevent insurance companies and employers from manipulating employees’ genetic information, hence leading to possible cases of genetic discrimination [78–81]. Additionally, the lack of stringent and well-established regulations on GT offered by commercial companies has also contributed to the heterogeneity of service quality and inaccuracy of test results [60]. Hence without a robust legal infrastructure to regulate the provision of GT as well as the manipulation and interpretation of results, Hong Kong undergraduates would remain concerned about the adverse impacts that an unfavorable GT could cast on employment and career options.

Apart from education to instill accurate knowledge, policymaking on the establishment of supporting services relevant to GT could increase public confidence and address psychological distress on an unfavorable testing result [82]. An evident illustration is the incorporation of genetic counselors to the human resources infrastructure of the healthcare system as implemented in most developed countries and proposed in the study by Mahmutovic et al. responding to the issue of pessimism [83, 84]. In pre-test counseling, genetic counselors could safeguard informed consent of GT and ensure psychological preparation on possible test results; in post-test counseling, they could disclose testing results, discuss future implications, and explore possible management plans, such as family cascade screening and disease surveillance, which are helpful to patients who have received unfavorable results [85–87]. Integrating supporting healthcare services like genetic counseling to complement existing practices provides an opportunity to resolve the apprehension of unfavorable GT results through a more multidisciplinary management.

Ultimately, by establishing a holistic ethical, legal, and social framework together with a strong and diverse network of supporting services on GT, future generations would be more optimistic to unfavorable GT result and be more confident and supportive of GT.

## Limitations

In this study, the participants were only recruited from one of the eight local universities by convenience sampling; hence, the results could not be generalized to all undergraduates or the general public in Hong Kong. Furthermore, there might be bias in data due to recruitment of participants from a course related to scientific and technological literacy, where undergraduates who enrolled in the course might be more interested in PM, PGx, and GT and/or equipped with more knowledge on related topics than general undergraduates.

However, despite the aforementioned limitations, this study provided evidence of the uniqueness in the perception of undergraduates on PM, PGx, and GT in Hong Kong, acting as a reference for future researches on the identification of current gaps in medical education and development of personalized medicine across the globe.

## Conclusions

This study illustrates the perception of PM, PGx, and GT among undergraduates in Hong Kong. It underscores the need of incorporating genomic medicine in education and career planning and highlights the importance of implementing PM by providing structured, comprehensive, and professional services on GT for citizens and enhancement of existing legal frameworks on various ELSI. With the implementation of holistic education and prudent policymaking in the future, Hong Kong is a step toward the era of genomic medicine.

## Supplementary Information

**Additional file 1.**

## Data Availability

The data used and/or analyzed during the current study are available from the authors upon reasonable request.

## Abbreviations

ADR: Adverse drug reactions
CPIC: The Clinical Pharmacogenetics Implementation Consortium
DTCGT: Direct-to-consumer genetic testing
DPWG: The Dutch Pharmacogenetics Working Group
ELSI: Ethical, legal, and social implications
ExAC: The Exome Aggregation Consortium
gnomAD: The Genome Aggregation Database
GT: Genetic testing
HKGP: Hong Kong Genome Project
IBM SPSS: IBM Statistical Package for Social Science
ISP: International Society of Pharmacogenomics
MRC: Medically related curriculum
NGS: Next-generation sequencing
NMRC: Non-medically related curriculum
PharmGKB: The Pharmacogenomics Knowledge Base
PGx: Pharmacogenomics
PM: Personalized medicine
