# Supplementary material for: Perception of personalized medicine, pharmacogenomics, and genetic testing among undergraduates in Hong Kong
Source: Hum Genomics. 2021 Aug 18;15:54. doi: 10.1186/s40246-021-00353-0 (PMC8371796; doi:10.1186/s40246-021-00353-0)
Supplement: Supplementary file 1 — Additional file 1. [file 40246_2021_353_MOESM1_ESM.pdf]

Perception of Personalized Medicine, Pharmacogenomics and Genetic Testing among undergraduates in Hong Kong

Supplementary Material: Questionnaire

1 Mark your age

<19

19-21

21-23

23-25

25-27

>27

2 Mark your gender

Male

Female

3 Describe your ethnic origin (e.g. Chinese – Sichuan)

4 Mark your year of study?

Year 1

Year 2

Year 3

Year 4

Others

5 What is your field of study?

Biomedical Sciences

Pharmacy

Nursing

Chinese Medicine

Medicine

Dentistry

Social Sciences

Business and Economics Education

Architecture Engineering

Law

Arts

Others

6 How would you rate your current level of knowledge in genetics?

I follow the latest research in genetics

I am quite familiar with basic concepts in genetics

I have learnt about genetics in school biology only

I know close to nothing about genetics

7 Have you been diagnosed with any of following diseases? You can choose multiple options.

Respiratory (asthma)

Psychiatry (depression, anxiety)

Cardiovascular (heart problems, atherosclerosis, hypertension)

Metabolic diseases (diabetes, metabolic syndrome)

Oncology (any type of cancer)

No

8 Did you ever take a drug that is used to treat any of the following diseases? You can choose multiple options.

Respiratory

Psychiatry

Cardiovascular

Metabolic disease

Oncology

Other

I do not take drugs

9 Have you ever had an adverse drug reaction?

Yes

No

Don't know

I have never taken any medication

10 Have you ever found that a particular drug did not work for you?

Yes

No

Don't know

I have never taken any medication

11 To what extent do you think that genes influence your health?

Completely

Moderately

Not at all

Don't know

12 Would you consider having a genetic test done to find out what illnesses you might develop in the future?

Yes

No

Don't know

13 Have you heard about personal genome testing companies?

Yes

No

Don't know Not sure

14 Would you consider contacting personal genome testing company and ordering a pharmacogenomic test for yourself?

Yes

No

Don't know Not sure

15 If you know your genetic tendency to develop a disease, would you be ready to make necessary changes in your lifestyle, to reduce disease risk?

Yes

No

Maybe

Don't know

16 If a pharmacogenomic test revealed that prescribed drug would either be ineffective or cause severe side effects, would you take the drug anyway?

Take the drug anyway

Accept the test result, and not take the drug

Accept the test result, and take the drug only if the disease might be

life-threatening Not sure

17 Do you agree that personalized medicine represent a new and promising healthcare model?

Yes

No

Don't know

18 Pharmacogenomics (the interaction between drugs and genetics) should be an important part of my study curriculum.

Agree

Disagree

Neutral

No opinion

19 Do you think that curriculum of your study program is well designed for understanding pharmacogenomics?

Not applicable Yes

No

Don't know

20 Would you like to continue your postgraduate education (master, PhD, specialization) in the field of personalized medicine?

Yes

No

Don't know

21 Which pharmacogenomics topics would you be interested to learn more about? You can choose multiple options.

Pharmacogenomics in general

Benefits of pharmacogenomics in clinical practices

Future development in pharmacogenomics

Clinical examples of pharmacogenomics

Ethical, legal and social issues related to pharmacogenomics

Pharmacogenomics has no relevance to me

22 How much money are you willing to spend to examine the effectiveness of a specific drug in your body using pharmacogenomic test? (Hong Kong Dollars)

<$100

$101 - $500

$501 - $1,000

$1,001 - $5,000

$5,001 - $10,000

$10,001 - $50,000

>$50,000

23 Are you aware of different ethical aspects of genetic testing?

Yes

No

Not sure

24 What ethical issues do you believe might be related to genetic or pharmacogenomic testing? Can choose multiple answers.

Patient privacy

Racial issues

Incidental findings

Data confidentiality

Stigma

Other

25 Are you worried about the possibility that the result of a pharmacogenomic test may be passed to unauthorized persons?

Not worried

Slightly worried

Very worried

I don't know

26 Which of the following healthcare professionals should have access to your pharmacogenomic information? You can choose multiple options.

Physician

Genetic counselor

Psychologist

Pharmacist

Social worker

Nurse

Nutritionist

27 Are you worried about the possibility that a pharmacogenomic test may reveal that you have additional risk factors for other diseases?

Not worried

Slightly worried

Very worried

No opinion

28 In case an unfavorable test result should be disclosed, do you believe that you would be disadvantaged at work or job-seeking?

Yes

No

No opinion

29 In case of an unfavorable test result, do you believe that you would feel “helpless” or “pessimistic”?

Yes

No

No opinion

30 In case of an unfavorable test result, do you believe that you would feel “different” or “inadequate”?

Yes

No

No opinion

31 Do you believe that in the future pressure may be exerted on patients to agree to perform a pharmacogenomic test?

Yes

No

No opinion

32 Do you consent for the anonymous use of responses provided for research and teaching development purposes?

Yes

No
